# About the Anti-Müllerian Hormone (AMH) Uses in the Clinical Practice

**DOI:** 10.1055/s-0038-1676059

**Published:** 2018-11

**Authors:** Gustavo Arantes Rosa Maciel, Edmund Chada Baracat, Marcos Felipe Silva de Sá

**Affiliations:** 1 Faculty of Medicine, Hospital das Clinicas HCFMUSP, Universidade de Sao Paulo, Sao Paulo, SP, Brazil; 2 Faculty of Medicine, Universidade de Sao Paulo, Ribeirão Preto, SP, Brazil

The anti-Müllerian hormone (AMH) is a glycoprotein that belongs to the transforming growth factor β (TGF-β) superfamily, and which has a key role in the male sexual development.^1^ It is produced by the Sertoli cells in the testis and induces the regression of Müllerian structures. In females, the roles of the AMH have been the focus of recent attention. In the present article, some aspects of the physiology, laboratory assessment, clinical uses and pitfalls, as well as current applications from the health perspective of women will be discussed briefly.

In humans, the AMH is encoded by the *AMH* gene, located on the chromosome 19p13.3, whereas the gene *AMHR2,* which codes for its receptor, is located on the chromosome 12.^1^ Although the physiology of the AMH in females is not completely established, a huge amount of data produced in the last few years have expanded the knowledge in this field.^1^ ^2^ This hormone is secreted by the ovarian granulosa cells of the preantral and small follicles. It has been demonstrated that the AMH plays an important role in the inhibition of the initiation of the primordial follicles.^2^ Data from *in vivo* and *in vitro* studies in animals and humans demonstrated that the AMH has a key role in the maintenance of the quiescent status of those follicles.^2^ Recently, it has also been shown that this inhibition might be induced by the administration of recombinant AMH or by an overexpression of the gene that encodes the ligand,^3^ opening a broad spectrum of possibilities for the clinical use of the AMH. Another important action in the ovary is that the AMH reduces the follicle sensitivity to follicle-stimulating hormone (FSH), depending on the stage of the follicular development.^4^ According to some authors, the AMH may be one factor that dictates in which stage the follicle will begin to respond to the FSH.^5^ In terms of antral follicles, it has been suggested that the AMH has a role in the selection process together with estradiol (E2) and inhibin.^6^ Furthermore, the AMH seems to downregulate the aromatase activity in the granulosa cells of follicles before selection.^7^ All of these functions in the antral follicles suggest that the AMH may act as a follicular gatekeeper and may ensure that each small antral follicle produces the adequate amount of E2 prior to the selection.^1^ ^8^ In 2016, an interesting study suggested that the AMH has a role in the gonadotropin-releasing hormone (GnRH) pulse generation control in the hypothalamus.^9^

The reference values of the AMH were established through several studies within the female lifespan.^10^ The interindividual variability of the AMH is high, mainly due to the highly variable number of follicles within groups of subjects of similar age.^11^ ^12^ Some features raise the attention for its clinical use: first, that AMH levels, after a peak around the second decade of life, decrease with age and are strongly correlated with the ovarian reserve. Second, the variability within the menstrual cycles, although present, is not significant from the clinical point of view.^12^ These features turned the assessment an appealing diagnostic tool.

To date, there are several possible uses for the AMH assessment: evaluation of the ovarian reserve (OR), prediction of controlled ovarian stimulation (COS), prediction of the natural age of menopause, assessment of the ovarian function, differentiation of some disorders of sex development (DSDs), and tumor marker, among others.^1^ ^12^ However, the approved uses in many countries (including Brazil) are an evaluation of the OR and the prediction of response to COS.^13^ ^14^ Thus, in the present review, due to space concerns, only the official approved indications will be discussed.

The most important use of AMH assessment is the evaluation of the OR. It is known that women are born with a given number of follicles (that harbor the female germ cells), and that most of these follicles will undergo atresia and will be depleted throughout their lifespan. A small portion will be ovulated. The loss of follicles is directly related with female fertility and, after 37 years of age, the rate of follicle death becomes more evident. The OR can be determined by the age of the woman, by the assessment of FSH and estradiol (E2), and by antral follicle counting (AFC) in the early follicular phase.^14^ ^15^ The AMH was included in 2002 as a tool of evaluation of the OR.^14^ ^15^ None of the OR markers should be used alone, because all of them present limitations.^15^ Due to the fact that the AMH reflects the number of preantral and small antral follicles, there is a relationship between the serum levels of AMH and the remaining follicles. The AMH also displays a strong correlation with the gold standard method of evaluation of the OR, which is AFC performed by sonography. A possibility of a more objective way to access the OR is an important tool for the clinical practice. To date, the AMH is considered the earliest and most sensitive marker of OR, especially in assisted reproductive technology (ART) scenarios.^15^ The main strength of AMH testing is to predict inadequate ovarian response, either poor ovarian response (POR) or excessive ovarian response. For instance, sensitivities range between 44 and 97%, and specificities range between 41 and 100%^14^ for a POR, if AMH levels are low (0.1–1.66 ng/mL).^16^ Anti-Müllerian hormone levels > 1.0 ng/mL but < 3.5 ng/mL, if the patient is in the appropriate age, are consistent with normal ovarian response to ovarian stimulation.^15^ However, in cases of natural fertility, the AMH seems to have a more limited predictability.^16^ ^17^ Assessing OR in the general population might add extra costs to the health system.^17^ However, showing an information about one's OR might lead individuals to modify life choices in terms of fertility decisions.^18^ ^19^ ^20^ The assessment of AMH levels is useful and reliable, but more studies shall further endorse the dissemination of its use.

Currently, the main indications for OR testing are applied in women undergoing infertility evaluation/treatment (including history of premature ovarian insufficiency, oocyte donation, fertility preservation due to social or personal issues or due to gonadotoxic treatments), prior to ovarian surgery in women in reproductive age, in polycystic ovary syndrome (PCOS), in perimenopause, and in women with mutation of *BRCA-*1 or *FMR1* premutation (Fragile X syndrome). In addition, OR assessment is useful in the individualization of ART ovarian stimulation.^15^

The second approved use of AMH assessment is the prediction of ovarian response in ART treatment.^14^ To date, it is well established that both AMH and AFC are strong predictors of ovarian response in in vitro fertilization (IVF).^21^ The measurement of AMH is useful in the prediction of poor response and cycle cancellation due to inadequate ovarian response, as well as in hyper-response and ovarian hyperstimulation syndrome, in COS.^14^ The AMH was shown to be a better marker in predicting ovarian response to COS than the age FSH, estradiol, and inhibin B.^14^ Recently, a study with a new human recombinant FSH (follitropin delta, rFSH), found that the AMH might be a tool in predicting an adequate ovarian response in COS, with lower doses of rFSH with similar number of blastocysts.^22^ However, another study, using a different AMH more studies are necessary to assess those findings in different platforms. It seems that AFC and AMH may have complementary roles in the preassessment of infertile women.^23^

An important issue for the clinical practice is related to technical aspects of the laboratorial assessment of the AMH serum levels. Several assays to detect the serum levels of the AMH were developed, but most of them are based on a sandwich type of immunometric or enzyme-linked immunosorbent assay (ELISA) tests with two monoclonal antibodies (ABs) that were both raised against recombinant human AMH (rhAMH). The ABs are able to recognize epitopes in the proregion (F2B/7A) and/or in the mature regions (F2B/12H).^24^ However, there have been studies questioning the stability of AMH upon storage, sample handling and sample diluting, due to a complement system interference, that could falsely alter the serum titles.^25^ This problem has motivated the biggest manufacturer of AMH tests to withdraw its tests from the world market in 2013, but a few assays remained available. In a short period of time, the manufacturers addressed the problem by adding the ABs in solution, instead of a in solid phase and new automated platforms were created. The complement interference issue seemed to be adequately solved.^23^ Although the implementation of automated platforms must be considered an advance, an international standard developed in accordance with the International Federation of Clinical Chemistry is still needed because, the variation amongst platforms can interfere in the clinical interpretation.^23^

Another issue for the diagnostic use of AMH is that its levels might be influenced by specific biological, reproductive or environmental conditions. The serum levels of AMH tend to be decreased in several clinical situations: low levels of vitamin D,^26^ use of oral contraceptive pills or GnRH agonists, endometriosis, endometriomas, history of ovarian surgery, smoking habit, mutations and permutation in the *BRCA-1* and in the *FMR1* genes, respectively. On the other hand, PCOS, granulosa cells tumors, and DSDs are associated with remarkable increases in the level of AMH.^23^ All these situations must be considered when AMH tests are used.

In conclusion, the assessment of AMH may be useful in several clinical situations, especially to evaluate the OR and to help to predict ovarian response in IVF cycles. However, an international standardization of the measuring methods is still necessary. Although an exciting amount of information is contributing to reveal its functions, the physiology of the AMH is not completely understood, and the elucidation of key steps of its physiological roles has the potential to increase its utility in the clinical practice.
